# Linked emergence of racial disparities in mental health and epigenetic biological aging across childhood and adolescence

**DOI:** 10.1038/s41380-025-03010-3

**Published:** 2025-04-09

**Authors:** Muna Aikins, Yayouk Willems, Deniz Fraemke, Colter Mitchell, Bridget Goosby, Laurel Raffington

**Affiliations:** 1 Max Planck Research Group Biosocial – Biology, Social Disparities, and Development; Max Planck Institute for Human Development, Berlin, Germany; 2 Survey Research Center of the Institute for Social Research; University of Michigan, Ann Arbor, MI USA; 3 Population Studies Center of the Institute for Social Research; University of Michigan, Ann Arbor, MI USA; 4 Department of Sociology and Population Research Center; University of Texas at Austin, Austin, TX USA

**Keywords:** Psychology, Biomarkers

## Abstract

Marginalization due to structural racism may confer an increased risk for aging-related diseases – in part – via effects on people’s mental health. Here we leverage a prospective birth cohort study to examine whether the emergence of racial disparities in mental health and DNA-methylation measures of biological aging (*i.e*., DunedinPACE, GrimAge Acceleration, PhenoAge Acceleration) are linked across childhood and adolescence. We further consider to what extent racial disparities are statistically accounted for by perinatal and postnatal factors in preregistered analyses of 4898 participants from the Future of Families & Child Wellbeing Study, of which 2039 had repeated saliva DNA methylation at ages 9 and 15 years. We find that racially marginalized children had higher levels of externalizing and internalizing behaviors and diverging longitudinal internalizing slopes. Black compared to White identifying children, children living in more racially segregated neighborhoods, and racially marginalized children more affected by colorism tended to have higher age-9 levels of biological aging and more biological age acceleration over adolescence. Notably, longitudinal increases in internalizing and externalizing behavior were correlated with increases in biological aging. While racial and ethnic disparities in mental health were largely statistically accounted for by socioeconomic variables, differences in biological aging were often still visible after including potential mediating variables. These findings underscore the urgency for future research to consider biological aging processes from early life and collect more comprehensive measures of structural racism in developmental cohorts. Programs dedicated to advancing racial health equity must address the psychological and physical effects of structural racism on children and adolescents.

## Introduction

A large body of evidence has recorded striking racial disparities in physical and mental health [1–6]. Therefore, examining how different manifestations of racism, including effects of institutionalized systems and interpersonal social dynamics in which individuals are “racialized”, affects health across the lifespan is a priority to improving population health [7–9]. For instance, heightened daily life stress and vigilance stemming from ongoing racialization may amplify the risk of poorer mental health and contribute to higher levels of chronic inflammation and accelerated multi-system biological aging [10–15]. Biological aging can be defined as the progressive loss of system integrity that occurs with advancing chronological age, including changes in DNA-methylation (DNAm; [16, 17]). DNAm measures of biological aging can be applied early in the life course to study the etiology of socially organized health disparities, decades before differences in disease and mortality are measurable [18].

Racial marginalization and low socioeconomic status has been associated with more advanced and faster biological aging as measured in DNAm in both adults and children, and, in adults, these differences in biological aging partially account for health disparities between and within racial groups [19–23]. A few studies have found DNAm measures of biological aging to be associated with mental health [21, 24, 25]. Yet, previous research has largely been cross-sectional in design and, thus, does not address the dynamic interplay between racialization, mental health, and biological aging.

Here we leverage a prospective birth cohort study to examine whether the emergence of racial disparities in mental health is linked to racial disparities in DNA-methylation measures of biological aging across childhood and adolescence. While previous studies have shown that individual- and family-level factors, such as family instability, are associated with child developmental and health outcomes [26–29], our analysis addresses an important gap in the literature by underscoring that macro-level racism reinforces health inequities at an early age. We conceptualized racial and ethnic disparities as outcomes of structural racism, more specifically, as “racialization”, which emphasizes the social processes and institutionalized systems in which individuals are positioned. First, we used self-identified race and ethnicity as an indicator of individuals’ racial social position. Importantly, race is a social construct, not a biological category, and the ways in which race is socially defined and experienced shape individuals’ access to resources and opportunities [30–33]. Second, we utilized the Thiel Index as a statistical measure that captures the extent of neighborhood racial segregation [34]. Racial segregation in US neighborhoods is largely a result of historical policies and practices such as redlining, discriminatory lending, and exclusionary zoning, which systematically marginalized communities [35]. Third, for racially marginalized youth who did not identify as solely White, we included skin tone as a proxy for colorism, recognizing that variations in skin color can influence social experiences and opportunities [34, 36–38]. Colorism perpetuates the racial hierarchy within marginalized communities as a consequence of historical conditions including colonialism, slavery, and the preferential treatment of lighter skin tones by dominant groups [36]. Moreover, we considered family-level and neighborhood-level socioeconomic conditions as well as perinatal (*e.g*., birthweight) factors, police interactions and parenting stress, as potential mediators of associations between structural racism and child development.

We probe these biosocial dynamics in preregistered analyses of N = 4898 participants from the Future of Families & Child Wellbeing Study, which intentionally oversampled financially under resourced families. Among the N = 2039 participants who had repeated saliva DNAm at ages 9 and 15 years, most participants racially positioned themselves as African-American/Black (*n* = 901, 47%), followed by Hispanic/Latinx (*n* = 511, 26%), White (*n* = 366, 19%), Multiracial (*n* = 99, 5%), and “Other” (*n* = 52, 3%).

## Methods

### Participants

The Future of Families and Child Wellbeing Study (FFCWS) follows a sample of 4898 children born in large US cities during 1998–2000. FFCWS oversampled children born to unmarried parents and interviewed parents at birth and ages 1, 3, 5, 9 and 15. During home visits, saliva DNA was collected the Illumina 450 K and EPIC methylation arrays with ages 9 (*n* = 1971) and 15 years (*n* = 1974) assayed on the same plate (for further information on DNA preprocessing see Supplemental Material). DNAm study participants self-identified race/ethnicity defined by study protocol as African-American/Black only (*n* = 901, 47%), “Other” (*n* = 52, 3%), Hispanic/Latinx (*n* = 511, 26%), Multiracial (*n* = 99, 5%), White (*n* = 366, 19%). The University of Michigan and Princeton University Institutional Review board granted ethical approval. Informed written consent was obtained from all participants and study participants’ legal guardians. All methods were performed in accordance with the relevant guidelines and regulations.

### Measures

#### Mental health

Parent-reported internalizing and externalizing behaviors were assessed at ages 3, 5, 9, and 15 years using the Child Behavior Checklist (CBCL) [39]. In addition, children self-reported symptoms of depression and anxiety at 15 years [40]. See Table 1 for a detailed description of these measures.

**Table 1:** Description of measures.

Variable	Description
Mental Health	
Parent-reported internalizing & externalizing behavior at 3, 5, 9, 15 years	We used the Child Behavior Checklist (CBCL) to assess internalizing and externalizing behaviors [39]. The CBCL is a parent reported assessment with items that measures behavioral problems for each age in a developmentally appropriate manner. Parents indicated whether the statement of the behavior in the last 6 months was “never true” (coded 0), “sometimes or somewhat true” (coded 1), or “very true or often true” (coded 2). A mean score was created across items, with higher scores indicating more internalizing or externalizing behaviors. The CBCL is a well validated and frequently used assessment in FFCW, showing good internal consistency and test-retest reliability (cf. [26, 71, 72]. In line with the codebook of FFCW, internalizing scores included items of the anxious/depressed, somatic complaints and withdrawn subscales and externalizing scores included items of the aggressive and rule-breaking/destructive subscales (see Supplemental Material Table 2 for an overview). We used mean scores to accommodate the difference in items across time, which has been supported by previous longitudinal studies in the FFCW [73]. Internalizing and externalizing scores were log-transformed to correct for significant skew.
Child-reported depressive symptoms at 15 years	We used items of the Center for Epidemiologic Studies Depression Scale [74] scale to assess self-reported depressive symptoms. The scale consisted of 5 items, with questions such as “I feel I cannot shake off the blues, even with help from my family and my friends”, “I feel sad”, “I feel depressed”. Adolescents were asked to rate these items about the past four weeks on a scale ranging from strongly agree (coded 1) to strongly disagree (coded 4). Items were re-coded such that a higher mean score reflects more depressive symptoms. The scale showed good internal consistency (Cronbach 〈 = 0.76).
Child-reported anxiety symptoms at 15 years	We used items of the Brief Symptom Inventory 18 [75] to assess anxiety symptoms in adolescents. The scale consisted of 6 items, with questions such as “did you in the last month “have spells of terror or panic,” “feel tense,” “feel nervous”. Response choices ranged between strongly agree (coded 0) to strongly disagree (coded 3). Items were re-coded, with higher mean scores reflecting higher anxiety symptoms. The scale showed good internal consistency (Cronbach 〈 = 0.76).
DNA methylation measures of biological aging	
DunedinPACE at 9, 15 years	The DunedinPACE metric was devised within the context of the Dunedin Study birth cohort, deriving from an examination of intra-individual fluctuations in 18 physiological indicators, which were assessed at multiple time points, specifically at the ages of 26, 32, 38, and 45 [41].
GrimAge Acceleration at 9, 15 years	GrimAge represents a DNAm metric designed to predict morbidity and mortality. Briefly, the initial phase entailed the computation of models incorporating physiological indicators, age, sex, and smoking history, with the objective of optimizing mortality prediction within the Framingham Heart Study Offspring cohort [42].
PhenoAge Acceleration at 9, 15 years	PhenoAge is conceptualized on the foundation of physiological markers and chronological age, which are subsequently employed to model a novel sample derived from DNA methylation, culminating in the establishment of a definitive DNA methylation clock [43]. This metric exemplifies the age, measured in years, at which the average mortality risk in the NHANES III cohort aligns with the mortality risk as forecasted by the PhenoAge algorithm. DNA methylation measures were residualized for cell composition using Epidish count of immune cells, Epidish count of fibroblasts and Array (450k or EPIC). Age 9 and 15 were run on the same batch. GrimAge and PhenoAge were additionally have principal components versions to bolster reliability (see [76] and residualized for chronological age derived from sample receipt age to reflect accelerated biological age. See “preprocessing DNA data” in the Supplemental Material for more details on the computation of these DNA methylation measures of biological aging.
Children’s race and ethnicity	
Child-reported Race and ethnicity at 15 years	Adolescents were asked to self-identify their race and ethnicity in verbatim responses, using up to 80 characters. Dummy variables were created by a committee of four staff members, resulting in the following categories: Black/African American and non-Hispanic, Hispanic/Latinx, multiracial and non-Hispanic, other race and non-Hispanic, and White and non-Hispanic. These were strongly tied to prior race reporting (years 0–9) by the parents.
Home visit interviewer-rated skin tone at 15 years	During home visits, the interviewer rated the skin color of the adolescent on an 11-point scale ranging from albinism (coded 0) to darkest possible skin (coded 10). The scale is based on the Massey and Martin Skin Color Scale with identical hands differing in colors. The scale was used by interviewers, who memorized the scale, so that the respondent never saw the chart.
Neighborhood-level racial, ethnic context	
Census data on racial segregation at 0 and 9 years	Multi-group Theil index calculated at the county level over four groups: White alone, Black alone, Hispanic, and Other. The Theil index measures the segregation of a particular group of people within a metropolitan area. This index spans from 0.0, indicating complete integration (when all areas have the same composition as the entire metropolitan area) to 1.0, indicating complete segregation (when all areas contain one group only) (see https://www.census.gov/topics/housing/housing-patterns/guidance/appendix-b.html). We averaged the Theil index across ages 0 and 9 years. Higher scores indicate living in a more segregated area.
Family-level SES	
Parent-reported household income at baseline, 9, and 15 years	Household income included total income from all sources earned before taxes in the household. At age 9 and age 15 there were a couple of families with incomes higher than 500.000 dollar (*n* = 3 at age 9, *n* = 5 at age 15). Deviating from the preregistration, we Windsorized these data points, capping them to 500.000 dollar to reduce the influence of these outliers on our statistical analyses without discarding these data points.
Parent-reported education level at baseline, 9, and 15 years.	Education level was categorized as: “some high school or less”, “high school diploma or GED”, “some college or 2-year degree”, “Bachelor’s degree”, “graduate school or higher”. We deviated from the preregistration in two ways: 1) In the preregistration we stated we would include parent-reported education level at baseline and age 9. Upon receiving the data, we realized there was also data available at age 15, just as for household income, which is why we included this age as well, 2) instead of including education level of both parents, we only included education level of mothers due to limited data availability of fathers (less then 40% at age 9, less then 7% at age 15). We created a family-level SES composite aggregating the average of standardized parent educational attainment and standardized, log-transformed household income. We averaged family-level SES across baseline, age 9 and age 15 into one overall family-level SES variable.
Neighborhood-level SES	
Census data on concentrated poverty at age 5, 9 and 15	Percent of families living below poverty threshold in the neighborhood where the child resides. We averaged this measure across age 5, 9 and 15.
Census data on public assistance at age 5, 9 and 15	Percent of households on public assistance in neighborhood where the child resides. We averaged this measure across age 5, 9 and 15.
Home visit interviewer-rated neighborhood conditions at age 5, 9 and 15	During home-visits, a researcher rated the neighborhood conditions on 5 dimensions such as conditions of buildings on the block (0 = well-kept with good repairs and exterior surfaces to 3 = badly deteriorated), if there is garbage, litter or broken glass on the street (0 = almost none to 3 = yes; almost everywhere) and if there are vacant, abandoned, or boarded-up buildings on the block or within 100 yards of their home (0 = no to 3 = yes; 4 or more buildings fit this description). Higher scores indicate worse neighborhood conditions. The scale showed good internal consistency (Cronbach 〈 = 0.85 at age 5, Cronbach 〈 = 0.78 at age 9, Cronbach 〈 = 0.79 at age 15). We averaged neighborhood conditions across age 5, 9 and 15. We averaged census data and neighborhood conditions as an indicator of neighborhood-level SES, with higher scores indicating more disadvantaged neighborhood. In the preregistration we stated we would average these three measures across baseline, age 9 and 15, but upon inspection of the data we realized data was available at age 5 not at baseline. As such, we included neighborhood level measures of poverty, public assistance and neighborhood conditions across age 5, 9 and 15.
Parenting	
Parent-reported parenting stress at 5, 9, 15 years	Parenting stress was assessed on a 4-point scale including items such as “being a parent is harder than I thought it would be”, “I often feel tired, worn out, or exhausted from raising a family”. The scale showed decent internal consistency (Cronbach 〈 0.66 at age 5, Cronbach 〈 = 0.78 at age 9, Cronbach 〈 = 0.68 at age 15). We averaged parenting stress across ages 5, 9, 15 years, with higher scores indicating more parenting stress. We included measures based on mother-report as for 88% of the children’s mothers were the primary caregivers (in 7% the father, 4% other relative, 1% non-relative being the primary caregiver).
Child-reported parent-child closeness at 15 years	Closeness between caregiver and child was assessed with the following two items: closeness between caregiver/child, degree to which caregiver/child talk and share ideas. Items were rated on a 4-point scale, with higher scores indicating less closeness. We re-coded these items, so that higher scores indicated more closeness. We included measures based on mother-report as for 88% of the children’s mothers were the primary caregivers (in 7% the father, 4% other relative, 1% non-relative being the primary caregiver). We deviated from our preregistration by only including parent-child closeness at age 15 instead of averaging across age 9 and age 15, as the correlation between age 9 and age 15 was low (r = 0.14, *p* < 0.01). Additionally, we did not include the item “the number of friends of the child the caregiver can identify” as this was not coded on the 4-point scale.
Police interactions	
Child-reported police interactions at 15 years	Interactions with police were measured with the following items: have you ever been stopped by the police 1) at school, 2) in your neighborhood, at 3) school, 4) or some other place (yes = 1, no = 0). Additionally, adolescents were asked if they have seen 5) parents, 6) siblings, 7) friends or neighbors being stopped by the police (yes = 1, no = 0). We measure child-reported police interactions with two items: direct police stops when ever been stopped by the police (“k6e10”) coded (yes = 1, no = 0) and vicarious police stops when reported knowing anyone stopped by the police (“k6e16”) coded (1 = yes, 2 = no) we recoded to (yes = 1, no = 0) so that higher scores mean having had interaction with the police.
Other Covariates	
Child- and parent-reported smoking at 15 years	Self-reported adolescent smoking and parent smoking were counted as true if they reported smoking at any measurement occasion (0 = never smoked, 1 = ever smoked).
Children’s body mass index (BMI) at 9, 15 years	BMI measurements were collected in participants’ homes by an interviewer, recording height and weight. These were transformed to sex- and age-normed z-scores according to the method published by the US Centers for Disease Control and Prevention (https://www.cdc.gov/growthcharts/percentile_data_files.htm).
Parent-reported pubertal development at year 9.	Pubertal development was assessed through parent report using the Pubertal Development Scale [77]. This scale includes questions across sex-specific domains (e.g., “have you noticed facial hair starting to grow” for boys, “child had first menstrual period” for girls) and general questions about teen’s pubertal development (e.g., “noticed that a child’s growth spurt started”). Initially, we preregistered to include pubertal development at year 15 as well, but excluded this from the analyses as this was not asked at this age.
Sex at birth	Sex at birth was reported by mothers at baseline (1 = boy, 2 = girl).
Medical record data on (pre)natal conditions	We included birth weight in grams, gestational age, and maternal use of drugs, alcohol and cigarettes during pregnancy. We did not preregister these variables, but included them in our analyses as these have been linked to DNAm aging [78].

#### DNA methylation measures of biological aging

The pace of biological aging was measured using DunedinPACE at 9, 15 years [41]. Accelerated biological aging was measured with GrimAge [42] and PhenoAge Acceleration [43]. See Table 1 for a detailed description.

#### Structural racism

We conducted a comprehensive analysis to quantify racism by employing three measures: self-identified race/ethnicity, the Thiel Index as a statistical measure that captures the extent of racial segregation within neighborhoods, and, for racially marginalized youth who did not identify as solely White, we included skin tone as a proxy for colorism, Moreover, we considered family-level and neighborhood-level socioeconomic factors, as well as police interactions and parenting stress, and perinatal factors as potential mediators of associations between racialization processes and child development. See Table 1 for a detailed description of these measures. See Supplemental Table 1 for descriptive statistics of measures of interest.

### Statistical analyses

Our preregistered analyses (https://osf.io/xbgzu) and results are categorized into three objectives. Supplemental Material Table 1 lists preregistered analyses and analytic deviations.

#### Racial and ethnic disparities in mental health from early childhood through adolescence

**Fig. 1:**
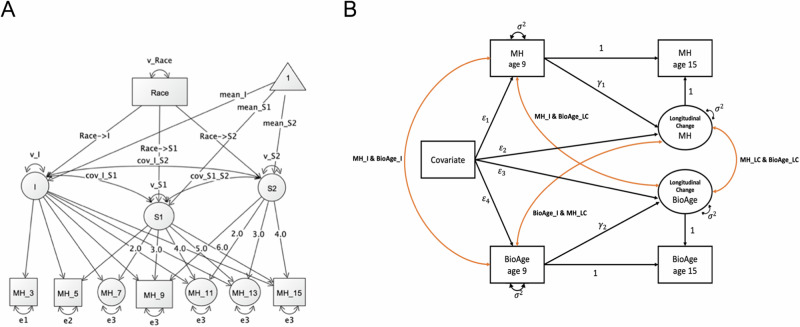
Longitudinal structural equation models. **A** depicts a latent growth curve model of mental health and race. Squares represent observed variables. Circles represent latent factors, including missing waves, intercepts, and slopes. Triangles denote constants, *i.e*. mean intercept and slopes (S1 = S_linear_; S2 = S_adolescence_). Single headed arrows denote regressions, and double headed arrows denote covariances. **B** depicts a latent change score model of mental health and DNA methylation measures of biological aging across adolescence. Squares represent observed variables. Circles represent latent factors. Single headed arrows denote regressions, and double headed arrows denote correlations. MH = Mental health measures of parent-reported externalizing and internalizing behaviors. BioAge = DNA-methylation measures of biological aging. I = Intercept. LC = Longitudinal change from 9-to-15-years. The estimated means of intercepts, longitudinal change, and covariates (*e.g*., prenatal factors) are not illustrated here, but were included in the model.

We examined racial and ethnic disparities in parent-reported internalizing and externalizing behaviors longitudinally across early childhood through adolescence, and in cross-sectional analyses of self-reported anxiety and depressive symptoms at age 15. Internalizing and externalizing scores were log-transformed. We applied a two-slope **latent growth curve model (LGCM)** depicted in Fig. 1A that estimated initial levels (intercepts) and rates of change over time (slopes) [44]. Latent factors were added for ages 7, 11, and 13 years to capture the trajectory of change in two-year intervals, accounting for missing observations [45, 46]. A two-slope LGCM is preferred over a one-slope model when the data exhibits different growth patterns over distinct periods and was found to improve model fit estimates (**see** Supplemental Table 3 and 4). The intercept (I) represents the estimated starting point or initial level of the outcome variable, for instance internalizing scores at the baseline age (*i.e*., at 3 years). S_linear_ represents the average linear change in the internalizing scores over childhood, *i.e*., as children age from 3 to 15 years. A negative mean S_linear_ indicates decreasing internalizing symptoms as children get older, on average. S_adolescence_ represents additional change in internalizing scores from 9 to 15 years. A positive mean S_adolescence_ indicates increasing internalizing symptoms over adolescence, on average. We regressed latent intercepts and slopes on measures of structural racism to examine how racial disparities in internalizing and externalizing trajectories evolved over time. Missing data was accounted for using full information maximum likelihood, and no participant was excluded from our analysis. We used robust maximum likelihood estimation to account for non-normality.

#### Racial and ethnic disparities in biological aging across adolescence

Next, we tested for racial and ethnic disparities in repeated measures of saliva DNAm quantifications of biological aging [25, 47, 48] in Latent Change Score (LCS) models depicted in Fig. 1B [49, 50]. Biological aging measures were standardized to baseline age levels at age 9. The model estimated initial levels, for instance racial disparities in mean biological age scores at 9 years, and latent changes, for instance racial disparities in mean longitudinal changes in biological aging from 9-to-15 years. We regressed initial levels and latent change on measures of structural racism to examine racial disparities in biological aging across adolescence.

#### Associations of mental health and biological aging

Lastly, we assessed if changes in biological aging from 9-to-15-years were correlated with changes in internalizing and externalizing behaviors from 9-to-15-years in LCS models (see Fig. 1B). We correlated initial levels in biological aging and mental health, which, for instance, may indicate that higher biological aging at age 9 years is associated with higher externalizing symptoms at age 9 years. We also correlated initial levels in biological aging and mental health with latent changes, which, for instance, may indicate that higher externalizing symptoms at age 9 years is associated with higher changes in biological aging from age 9-to-15 years. We also correlated latent changes which, for instance, may indicate that higher changes in biological aging from age 9-to-15-years is associated with higher externalizing symptoms from age 9-to-15-years.

We report nominal *p*-values with an alpha < 0.05 threshold and note if results remain significant after Benjamini-Hochberg False-Discovery-Rate correction (FDR, [51]. An overview of all our FDR corrections can be found in Supplemental Table 16 and 17. All data used in the present study is available to eligible researchers via FFCWS management (https://ffcws.princeton.edu/documentation). Examples of Mplus scripts for the main models can be found in the supplemental material, with pre-processing scripts in R available through our GitLab repository upon request.

## Results

### Racial and ethnic disparities in mental health from early childhood through adolescence

#### Externalizing behaviors

**Fig. 2:**
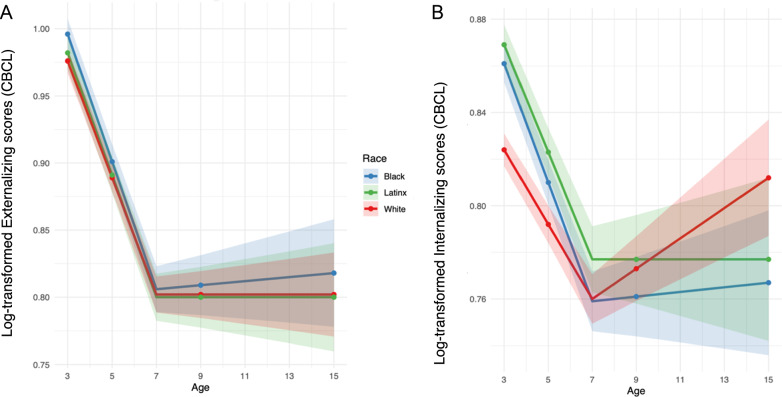
Racial and ethnic disparities in externalizing and internalizing behaviors from early childhood through adolescence. **A** and **B** depict model-predicted trajectories in log-transformed parent-reported externalizing and internalizing behaviors, respectively, for Black, Latinx and White children. Data for the smaller subsamples of Multiracial and Other are not shown for visualization purposes but were included in the analyses. Error bars depict the sum of the standard errors of the intercept and slope estimates. See Supplemental Material Fig. 3 for similar plots of observed mean scores.

First, we examined associations of self-identified race/ethnicity, neighborhood segregation, and skin tone with externalizing behaviors in the full sample of N = 4898. As depicted in Fig. 2A, we found that Black and Multiracial children compared to White identifying children had higher parent-reported externalizing levels across early childhood through adolescence (Black: *b* = 0.09, 95%CI [0.03, 0.16], *p* < 0.01, Multiracial: *b* = 0.06, 95%CI [0.01, 0.11], *p* < 0.05, significant after FDR correction). Racial disparities in slopes and interactions of race with gender did not remain significant after FDR correction (Supplemental Table 3; Supplemental Material Fig. 1).

Further, children living in more racially segregated neighborhoods, (who were more likely to identify as Black and Multiracial, Supplemental Material Figure 4), had a higher externalizing intercept (*b* = 0.09, 95%CI [0.04, 0.13], *p* < 0.05, significant after FDR correction) and a steeper decline over early childhood (*b* = –0.08, 95%CI [–0.15, –0.01], *p* < 0.05, significant after FDR correction, Supplemental Table 5). Amongst Black, Latinx and Multiracial children, darker skin tone was associated with a higher externalizing intercept (*b* = 0.09, 95%CI [0.01, 0.18], *p* < 0.05, significant after FDR correction, Supplemental Table 6).

#### Internalizing behaviors

Second, we examined associations of race/ethnicity, neighborhood segregation, and skin tone with **internalizing behaviors**. As shown in Fig. 2B we found that all groups of racially marginalized children showed higher internalizing levels across childhood compared to White children (Black *b* = 0.27, 95%CI [0.20, 0.34], *p* < 0.001; Latinx *b* = 0.28, 95%CI [0.21, 0.35], *p* < 0.001; Other *b* = 0.06, 95%CI [0.01, 0.12], *p* < 0.05; Multiracial *b* = 0.12, 95%CI [0.06, 0.18], *p* < 0.001, significant after FDR correction, Supplemental Table 4). Moreover, racially marginalized children had a steeper decrease in internalizing behavior compared to White children (S_linear_ in Supplemental Table 4; significant after FDR correction). The subsequent increase in internalizing behaviors from age 9-to-15 years did not differ significantly across groups (all *p* > 0.05).

There was some evidence that racial disparities in internalizing symptoms differed by gender (interaction race and gender S_adolescence_ *b* = –0.44, 95% CI [–0.77, –0.12], *p* < 0.01, significant after FDR correction). Accordingly, Black compared to White boys had a steeper increase in internalizing symptoms across adolescence. Conversely, Black and Hispanic girls displayed an attenuated increase in internalizing symptoms compared to White girls across adolescence (Supplemental Table 4; Supplemental Material Fig. 2B).

Further, children living in more racially segregated neighborhoods had a higher internalizing intercept (*b* = 0.07, 95%CI [0.02,0.12], *p* < 0.001, significant after FDR correction), and a steeper decline in internalizing behavior across childhood (S_linear_ *b* = –0.12, 95%CI [–0.20, –0.04], *p* < 0.01, significant after FDR correction, Supplemental Table 5). Amongst Black, Latinx and Multiracial children, darker skin tone was not significantly associated with internalizing behaviors (Supplemental Table 6).

#### Covariate analyses

Next, we assessed to what extent these racial/ethnic disparities in child mental health were statistically accounted for by covariate adjustment for proximal contextual factors related to structural racism (*e.g*., family socioeconomic status, neighborhood socioeconomic disadvantage, police interactions, parenting stress and closeness).

Racial and ethnic disparities in externalizing and internalizing behaviors were largely statistically accounted for by covariate control for family socioeconomic status and neighborhood socioeconomic disadvantage, whereas covariate control for police interactions and parenting had little effect (Supplemental Tables 3–6). Importantly, all groups of racially marginalized children were far more likely to live in socioeconomically under resourced families and neighborhoods, whereas age-15 adolescent reports of police interactions and parenting stress showed race differences for Black compared to White children only, with Black children reporting higher levels of direct police interaction and their parents reporting higher stress (Supplemental Table 2 and Supplemental Material Figure 4).

#### Anxiety and depressive symptoms

We did not find evidence of racial and ethnic disparities in self-reported anxiety or depressive symptoms at age 15 (Supplemental table 7). Amongst marginalized youth only, darker skin tone was associated with more anxiety symptoms (*b* = –0.07, 95%CI [–0.14 to –0.01], *p* < 0.05), but this result did not survive FDR correction (Supplemental table 7).

### Racial and ethnic disparities in biological aging across adolescence

**Fig. 3:**
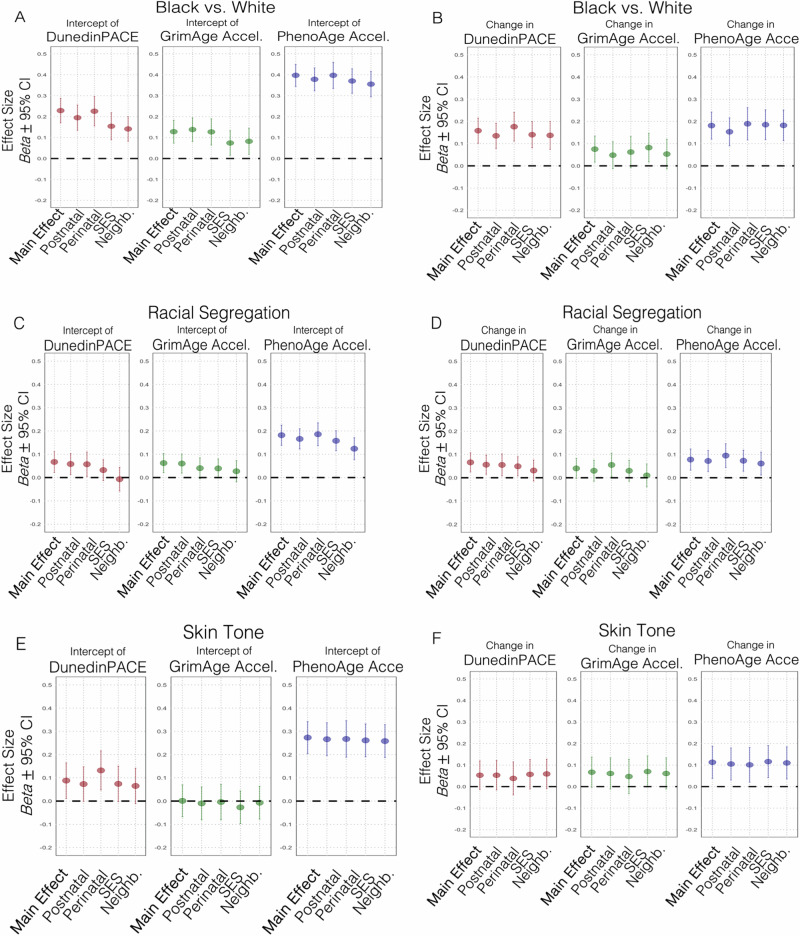
Racial and ethnic disparities in DNA-methylation measures of biological aging across adolescence. **A** depicts the difference in biological aging age-9 intercepts (DunedinPACE, GrimAge Acceleration, PhenoAge Acceleration) between Black compared to White identifying children without (i.e., Main Effect) and with covariate adjustment (covariates: postnatal factors, perinatal factors, family socioeconomic status [SES], and neighborhood disadvantage [Neighb.]). **B** depicts the difference in longitudinal change from age-9-to-15 in biological aging between Black compared to White children without and with covariate adjustment. **C** and (**D**) depict associations of neighborhood racial segregation with biological aging intercepts and longitudinal change, respectively. **E** and (**F**) depict associations of skin tone with biological aging intercepts and longitudinal change, respectively. Plotting the main effect and the covariates shows that the associations between racialization (self-identified race/ethnicity, racial segregation and skin tone, respectively) and biological aging were only slightly reduced (change in standardized effect sizes +/− 0.01–0.05) and remained statistically significant after accounting for perinatal (gestational age, birthweight, substance use during pregnancy) and postnatal (BMI, smoking, puberty status) covariates. Associations between racialization and biological aging were more substantially reduced after accounting for SES and neighborhood disadvantage.

We tested for associations of race/ethnicity, neighborhood segregation, and skin tone with biological aging measured at age 9 and 15 years in N = 2039 children with DNAm (see Fig. 1B for a graphical model illustration). We found that Black and Latinx compared to White youth tended to have a higher intercept and higher longitudinal increase in the pace of aging (DunedinPACE) across adolescence (Fig. 3A & B, Supplemental Table 8, significant after FDR correction). Black compared to White children also had a more advanced biological age intercept and higher longitudinal increase in biological age (GrimAge and PhenoAge Acceleration; Supplemental Table 8, Fig. 3, significant after FDR correction). Further, children living in more racially segregated neighborhoods had a higher intercept in all measures of biological aging, and a higher longitudinal increase as indicated by DunedinPACE and PhenoAge Acceleration (Fig. 3C, D; Supplemental Table 9, significant after FDR correction).

Amongst Black, Latinx and Multiracial children, darker skin tone was associated with a faster pace of aging (DunedinPACE) intercept and more advanced biological age intercept and higher longitudinal increase in biological age as indicated by PhenoAge Acceleration (Supplemental Table 10, Fig. 3E, F, significant after FDR correction). No significant associations were found for skin tone and GrimAge Acceleration (Supplemental Table 10).

Subsequently, we tested to what extent these associations were statistically accounted for by covariate adjustment for factors previously associated with DNAm measures of biological aging and/or structural racism, such as postnatal covariates (BMI, smoking, puberty status), perinatal birth factors (gestational age, birthweight, substance use during pregnancy), as well as proximal contextual factors (family socioeconomic status, neighborhood disadvantage, parental stress, parental closeness, and police interactions). The difference in biological aging between Black and White youth in biological aging largely persisted after accounting for postnatal covariates, perinatal birth factors, as well as proximal contextual factors (Fig. 3A, B, Supplemental Table 8). Associations of neighborhood racial segregation with biological aging largely persisted after accounting for postnatal, perinatal, police and parenting factors, but associations with DunedinPACE and GrimAge Acceleration were fully accounted for by socioeconomic status and neighborhood disadvantage (Fig. 3C, D, Supplemental Table 9). The association between skin tone and PhenoAge Acceleration remained significant after accounting for all covariates including socioeconomic status and neighborhood disadvantage, while its association with DunedinPACE was accounted for by postnatal, SES and neighborhood effects (Supplemental Table 10, Fig. 3E, F).

### Associations of mental health and biological aging

First, we examined whether longitudinal change from age-9-to-15-years in externalizing behaviors was associated with longitudinal changes from age-9-to-15-years in biological aging (see Fig. 1B for a graphical model and Table 2 for parameter estimates). We found that higher longitudinal increases in externalizing behavior were positively correlated with in pace of aging and biological age acceleration (DunedinPACE: *r* = 0.06, 95%CI [0.03, 0.13], *p* < 0.01; GrimAge Acceleration: r = 0.06, 95%CI [0.02, 0.11], p < 0.01; PhenoAge Acceleration: *r* = 0.05, 95%CI [0.01, 0.10], *p* < 0.05; significant after FDR correction). These associations largely persisted after accounting for perinatal, postnatal covariates, SES and neighborhood disadvantage (Supplemental Table 11; see Supplemental Table 12 for longitudinal correlations from subgroup analysis of White, Black and Latinx groups).

**Table 2:** Parameter Estimates.

		Externalizing					Internalizing				
BioAge	Parameters	*r*	SE	*p*	95% CI		*r*	SE	*p*	95% CI	
DunedinPACE	MH_I & BioAge_I	0.02	0.02	0.48	−0.03	0.06	0.04	0.02	0.12	−0.01	0.09
	MH_I & BioAge_LC	**0.07**	**0.02**	**0.00**	**0.03**	**0.12**	0.03	0.02	0.20	−0.02	0.08
	BioAge_I & MH_LC	−0.01	0.02	0.66	−0.06	0.04	−0.03	0.02	0.22	−0.07	0.02
	MH_LC & BioAge_LC	**0.06**	**0.02**	**0.01**	**0.03**	**0.13**	**0.06**	**0.02**	**0.01**	**0.01**	**0.10**
GrimAge Acceleration	MH_I & BioAge_I	**0.05**	**0.02**	**0.04**	**0.01**	**0.09**	**0.06**	**0.02**	**0.01**	**0.02**	**0.11**
	MH_I & BioAge_LC	**0.05**	**0.02**	**0.04**	**0.01**	**0.09**	−0.01	0.03	0.59	−0.06	0.04
	BioAge_I & MH_LC	0.02	0.02	0.45	−0.03	0.06	−**0.06**	**0.02**	**0.01**	−**0.11**	−**0.02**
	MH_LC & BioAge_LC	**0.06**	**0.02**	**0.01**	**0.02**	**0.11**	0.04	0.02	0.10	−0.01	0.08
PhenoAge Acceleration	MH_I & BioAge_I	**0.06**	**0.02**	**0.01**	**0.01**	**0.11**	0.03	0.02	0.20	−0.02	0.08
	MH_I & BioAge_LC	0.02	0.02	0.41	−0.03	0.07	−0.06	0.02	0.02	−0.11	−0.01
	BioAge_I & MH_LC	0.02	0.02	0.47	−0.03	0.06	−0.06	0.02	0.01	−0.10	−0.02
	MH_LC & BioAge_LC	**0.05**	**0.02**	**0.02**	**0.01**	**0.10**	**0.06**	**0.02**	**0.01**	**0.01**	**0.10**

Longitudinal changes in externalizing and internalizing behaviors with longitudinal changes in biological aging from 9-to-15-years.

Parameters correspond to path labels in Fig. 3, *MH_I & BioAge_I* = correlation between mental health intercept at age 9 and biological age intercept at age 9, *MH_I & BioAge_LC* = correlation between Intercept mental health at age 9 and longitudinal change in *BioAge* from age 9-15-years*, BioAge_I & MH_LC* = correlation between Intercept DNAm at age 9 and longitudinal change in mental health from age 9-15-years, *MH_LC & BioAge_LC* = correlation between longitudinal change in mental health and longitudinal change in BioAge from age 9-15-years. Significant associations at *p* < 0.05 are marked in bold. These associations hold after FDR correction.

Second, we tested whether longitudinal changes in internalizing behaviors were associated with longitudinal changes in biological aging. Higher longitudinal increases in internalizing behavior were correlated with higher longitudinal increases in pace of aging and biological age acceleration (DunedinPACE: *r* = 0.06, 95%CI [0.01, 0.10]*, p* < 0.05, PhenoAge Acceleration: *r* = 0.06, 95%CI [0.01, 0.10], *p* < 0.05, significant after FDR correction; GrimAge Acceleration: *r* = 0.04, 95%CI [0.01, 0.08], *p* = 0.08). These associations largely persisted after accounting for postnatal covariates, perinatal birth factors, SES and neighborhood disadvantage (Supplemental Table 11).

Lastly, we tested if age-15 biological aging was associated with mental health from early childhood through adolescence. We found more advanced biological age at age 15 years, as indicated by GrimAge and PhenoAge Acceleration, was associated with a higher externalizing intercept (GrimAge: *b* = 0.12, 95%CI [0.07, 0.18], *p* < *0.01*; PhenoAge: *b* = 0.07, CI [0.13, 0.12], *p* < 0.05), stronger decrease in childhood externalizing (GrimAge *b* = –0.14, 95%CI [–0.23, –0.04], *p* < 0.01), and a subsequently stronger increase over adolescence (GrimAge *b* = –0.20, 95%CI [–0.06, –0.03], *p* < 0.01), which remained significant after FDR correction. While the association with PhenoAge Acceleration was fully accounted for by socioeconomic variables, the association with GrimAge Acceleration largely remained significant after accounting for covariates (Supplemental Table 13). More advanced biological age, as indicated by GrimAge Acceleration, was also correlated with a higher internalizing intercept (*b* = 0.08, 95%CI [0.02, 0.15], *p* < 0.05; significant after FDR correction). This association was largely accounted for by postnatal covariates as well as family and neighborhood socioeconomic factors (Supplemental Table 14). A faster DunedinPACE-pace of aging at age-15-years was associated with higher concurrent levels of anxiety and depressive symptoms, and the association with anxiety symptoms remained significant after FDR correction as well as after covariate controls (anxiety: *b* = 0.07, 95%CI [0.02, 0.11], *p* < 0.01; depression: b = 0.05, 95%CI [0.01, 0.09], *p* < 0.05, Supplemental Table 15). An overview of all our FDR corrections can be found in Supplemental Tables 16 and 17.

## Discussion

We leveraged a prospective birth cohort study to examine whether the emergence of racial disparities in mental health is linked to the emergence of racial disparities in DNAm measures of biological aging across childhood and adolescence. We find that children who identify as part of racially marginalized groups and those residing in racially segregated neighborhoods exhibit higher levels of both externalizing and internalizing behaviors. Longitudinal trends in internalizing behaviors differed by race and gender. Moreover, Black children, compared to their White counterparts, as well as children from more racially segregated neighborhoods and those more strongly affected by colorism, tended to have higher levels of biological aging at age 9 and exhibit greater biological age acceleration during adolescence. Notably, increases in internalizing and externalizing behaviors over time were correlated with increases in biological aging. While socioeconomic factors largely statistically accounted for racial and ethnic disparities in mental health, racial differences in biological aging often persisted after controlling for these variables. This suggests that racial differences in mental health and biological aging manifest early in life and are interconnected.

Our findings are consistent with previous studies that have examined racial disparities in internalizing and externalizing behaviors among children and adolescents, as well as psychopathology in adults [26, 29]. While earlier research of this cohort has demonstrated that family-level factors, such as family instability and stress, are associated with child developmental and health outcomes [26–29], our analysis underscores the role of macro-level factors, particularly structural racism, in perpetuating health inequities from an early age. Additionally, our findings reveal that racial trajectories in mental health vary by gender, underscoring the need for future studies to consider the combined effects of race and gender on health [52].

Furthermore, our analysis of racial neighborhood segregation indicates that children living in more segregated neighborhoods, who were more likely to identify as Black and Multiracial, exhibit higher levels of externalizing and internalizing symptoms compared to their peers in more racially integrated neighborhoods. Our examination of skin tone amongst marginalized youth reveals that children facing greater social disadvantages related to skin tone (i.e., darker skin tone) tend to display higher externalizing behaviors. Individuals with darker skin tones often encounter a heightened risk of poor health outcomes compared to those with lighter skin tones [53, 54]. These racial health disparities arise from the racialization of darker-skinned phenotypes (colorism), a racial hierarchy that leads to marginalization and discrimination [36, 37]. This highlights the significant, yet understudied, role of skin tone as a social determinant of mental health.

Moreover, our study provides the first comprehensive analysis of structural racism and DNAm measures of biological aging in children. We found that Black compared to White identifying children, children living in more racially segregated neighborhoods, and marginalized children with darker skin tones, tended to have higher age-9 levels of biological aging and, importantly, more biological age acceleration over adolescence. These findings extend previous cross-sectional studies that have examined disparities related racialization in both children and adults [48, 55–57]. For instance, Hicken and colleagues [58] find that Black compared to White identifying adults have higher blood-based GrimAge and PhenoAge Acceleration (GrimAge Acceleration: *b* = 0.42, 95%CI [0.20, 0.64], *p* < 0.001; PhenoAge Acceleration: *b* = 0.29, 95%CI [0.02, 0.57], *p* < 0.001). Our saliva-based biological aging findings for 9-year-old Black children compared to White children are partially of a similar magnitude to these reports in adults (GrimAge Acceleration: *b* = 0.13, 95%CI [0.07, 0.18], *p* < 0.001; PhenoAge Acceleration: *b* = 0.40, 95%CI [0.34, 0.45], *p* < 0.001).

Lastly, we found that longitudinal increases in biological aging across adolescence were correlated with increases in internalizing and externalizing behavior. This is consistent with the interpretation that poor well-being has negative physical health consequences and vice versa [59, 60]. Alternatively, other factors, such as heightened racialized daily life stress and vigilance could concurrently influence both within-person change in mental health and biological aging over adolescence [10, 13]. These findings support the notion that racial disparities in biological aging result from early-onset and accumulating racialized experiences linked to biological stressors, and potentially highlight adolescence as a sensitive developmental period for lifespan health trajectories [60–64]. Over time, an increased mental health burden could contribute to racial disparities in disease and mortality, alongside unequal access to healthcare and educational opportunities [58].

Associations between mental health and racialization as well as between biological aging and neighborhood racial segregation and skin tone were largely statistically accounted for by socioeconomic variables. In contrast, associations between racialization and both levels and change in biological aging were only slightly attenuated and largely remained statistically significant after accounting for perinatal and postnatal covariates (e.g. birthweight, BMI). Structural racism creates socioeconomic advantages for some racial groups and disadvantages for others [65]. Accordingly, marginalized children are more likely to live in under-resourced families and neighborhoods: Black and Latinx children were 82.8% and 43.2% more likely, respectively, to live in disadvantaged neighborhoods compared to White children. Hence, separating racialized and socioeconomic inequality in racially stratified populations is statistically challenging and perhaps theoretically futile. Progress in understanding structural racism and health will come from applying intersectional perspectives and collecting dynamic measurements on racialization, such as economic health benefits varying across racial groups and measures of experienced racial discrimination, which are currently lacking [66]. Regular exposure to discriminatory policies and actions, especially in low-income, racially segregated areas, contributes to the emergence of racial disparities in physiological and psychological burden [9, 67, 68]. Our results substantiate neighborhood racial segregation as a health-relevant measure in childhood that is correlated with race/ethnicity and socioeconomic factors.

We note three major limitations of this study. First, despite our efforts to provide comprehensive measures of structural racism, the available metrics lack the nuances needed to capture both macro-structural and individual-level processes of racialization. Our findings underscore the urgent need for differentiated and comprehensive measures of racism in child developmental cohorts. Second, the study of racial disparities in biological aging is challenged by a lack of diversity in the discovery studies used to develop biological aging algorithms [18]. These studies predominantly involve White adults from higher-income countries, potentially leading to biased results. However, previous research in adults suggests that DNAm measures of aging are broadly applicable across human populations. For example, they consistently correlate with health and mortality across various countries [69] and are not associated with the percentage of European genetic admixture in non-Hispanic Black veterans [70]. Despite these findings, it remains crucial to develop biological aging algorithms that are inclusive and representative of the aging processes in diverse populations, highlighting a significant area for improvement in this field.

By applying DNA methylation algorithms, originally developed for adult studies on multi-system health and mortality, to children, our study reveals that the connection between mental and physical health—both of which are influenced by racism—emerges within the first two decades of life. It is imperative that programs dedicated to advancing racial health equity confront the psychological and physical repercussions of structural racism on children and adolescents.

## Supplementary information

Supplemental Tables

Supplemental Material

## Data Availability

This study utilizes data from the **Future Families and Child Wellbeing Study (FFCWS)**. Researchers interested in accessing the data can find detailed information on the **FFCWS website** at https://ffcws.princeton.edu, where additional documentation is available at https://ffcws.princeton.edu/documentation. Publicly available, de-identified data from the previous six waves of data collection can be accessed through the Princeton University Office of Population Research (OPR) Data Archive at https://ffcws.princeton.edu/data-and-documentation/public-data-documentation. For researchers requiring restricted-use contract data, access is granted only to those who agree to the terms outlined in the **FFCWS Contract Data License**. Further details on obtaining restricted data can be found at https://ffcws.princeton.edu/restricted.
