# Supplementary material for: Linked emergence of racial disparities in mental health and epigenetic biological aging across childhood and adolescence
Source: Mol Psychiatry. 2025 Apr 9;30(9):4296–306. doi: 10.1038/s41380-025-03010-3 (PMC12339396; doi:10.1038/s41380-025-03010-3)
Supplement: Supplementary file 2 — Supplemental Material [file 41380_2025_3010_MOESM2_ESM.docx]

Supplemental Material

[Supplemental Material Table 1 2](#_Toc190852133)

[Supplemental Material Table 2 8](#_Toc190852134)

[Preprocessing DNA data 14](#_Toc190852135)

[Supplemental Material Figure 1 15](#_Toc190852136)

[Supplemental Material Figure 2 16](#_Toc190852137)

[Supplemental Material Figure 3 17](#_Toc190852138)

[Supplemental Material Figure 4 18](#_Toc190852139)

[Examples of Mplus Scripts 19](#_Toc190852140)

[References 24](#_Toc190852141)

## **Supplemental Material Table 1**

| **Supplemental Material Table 1.** List of preregistered analyses (see https://osf.io/5sejf/), deviations, and results if not reported in main text. | | | |
| --- | --- | --- | --- |
| **Research Question** | **Preregistered analysis** | **Deviation from preregistration, if applicable** | **Reporting of the result / result if not reported in the main text** |
| 1. **Associations of race and mental health** | | | |
| Are children’s race/ethnicity and neighborhood racial segregation associated with mental health? Is this association moderated by socioeconomic status? | We use latent growth curve models of internalizing/externalizing intercepts (age 3 year) and slopes (change over time from 3 to 15 years) as well as in cross-sectional analyses of age-15 anxiety and depression. |  | See result section and **Supplemental Table 3, 4** (for longitudinal analyses) and **Supplemental Table** **7** (for cross-sectional analyses). |
|  | Regress mental health on children’s race/ethnicity. |  | See result section and **Supplemental Table 3 and 4** (for longitudinal analyses) and **Supplemental Table** **7** (for cross-sectional analyses). |
|  | Regress mental health on children’s race/ethnicity, socioeconomic contexts (family level SES and neighborhood disadvantage), and an interaction of children’s race/ethnicity by socioeconomic contexts. | In the pre-registration, we refer to socioeconomic contexts for both family and neighborhood SES. In our manuscript, we call this family level SES and neighborhood disadvantage to enhance clarity. | See result section and **Supplemental Table 3 and 4.** There were no significant neighborhood *race/ethnicity interactions, there was a significant family level SES* race interaction, for externalizing of LatinX Children. See **Supplemental Table 3.** |
|  | Regress mental health on neighborhood racial segregation. |  | See result section and **Supplemental Table 5.** |
|  | Regress mental health on neighborhood racial segregation, socioeconomic contexts (family level SES and neighborhood disadvantage), and an interaction of neighborhood racial segregation by socioeconomic contexts (family level SES and neighborhood disadvantage). | In the pre-registration we refer to racial context, in the manuscript to neighborhood racial segregation to enhance clarity. | There were no significant interaction effects of racial contexts by family or neighborhood SES for internalizing and externalizing. |
| To what extent do police interactions and socioeconomic contexts account for racial/ethnic disparities in mental health? | For significant associations, regress mental health on children’s race/ethnicity and police interactions. |  | See result section and **Supplemental Table 3 and 4.** |
|  | For significant associations, regress mental health on children’s race/ethnicity and socioeconomic contexts (family level SES and neighborhood disadvantage), |  | See result section and **Supplemental Table 3 and 4.** |
| Is skin tone associated with mental health amongst racialized youths? Is this association moderated by race/ethnicity? | For African-American/Black, Hispanic/Latinx, and Multiracial participants only, regress mental health on skin tone. |  | See result section and **Supplemental Table 6.** |
|  | For African-American/Black, Hispanic/Latinx, and Multiracial participants only, regress mental health on skin tone, race/ethnicity (African-American/Black, Hispanic/Latinx, and Multiracial) and an interaction of skin tone by race/ ethnicity. |  | There were no significant interaction effects of skin tone by race/ethnicity on mental health. |
| Does parenting moderate associations of racial/ethnicity with mental health? | Regress mental health on children’s race/ethnicity and parenting and an interaction of parenting by race/ethnicity. |  | See result section and **Supplemental Table 3 and 4**. Only significant interaction for externalizing; race by closeness for Multiracial Slope 2 (b = -1,024, CI =-1,861 to -0,187, p=0,016) |
|  | Regress mental health on neighborhood racial segregation, parenting, and an interaction of race by parenting. |  | See result section and **Supplemental Table 5**. There were no significant interaction effects of neighborhood racial segregation by parenting on mental health. |
| Does gender moderate associations of race/ethnicity with mental health? | Regress mental health on children’s race/ethnicity, gender, and an interaction of race by gender. |  | There was a significant race*sex interaction for Black children on Slope 1 and Slope 2 for Externalizing and Internalizing behavior, with more prominent effects for Black Boys (See results section and **Supplemental Table 4).** |
|  | Regress mental health on neighborhood racial segregation, gender, and an interaction of neighborhood racial segregation by gender. |  | There were no significant racial segregation * sex interactions. |
| 1. **Associations of race and DNAm-aging** | | | |
| Are race/ethnicity associated with DNAm-aging measured at age 9 and age 15? Is this association moderated by socioeconomic contexts? | Regress DNAm-aging on children’s race/ethnicity, separately for ages 9 and 15. | Instead of modeling cross-sectional effects, we applied a latent change score to investigate the effects of race/ethnicity on both the intercept and the change (i.e., delta). We deviated from the preregistration as this analysis allowed us to include the strength of the longitudinal data, and to gain better insights into developmental patterns. | See result section and **Supplemental Table 8.** |
|  | Regress DNAm-aging on children’s race/ethnicity, socioeconomic contexts (family level SES and neighborhood disadvantage), and an interaction of children’s race/ethnicity by socioeconomic contexts, separately for ages 9 and 15. | See above | See result section and Supplemental Table 8. There was no significant Family SES* race interaction on biological aging measures.  There was a significant neighborhood disadvantage*race interaction on the intercept of GrimAge acceleration and the longitudinal change of DunedinPACE (see **Supplemental Table 8,** only the interaction with the longitudinal change of Dunedinpace was significant after FDR correction).  The association between neighborhood disadvantage and biological aging was more prominent for White children than for marginalized children, although this did not hold FDR correction (intercept GrimAge Acceleration: White b=.19, 95%CI=.10 to .27, p<.001, Black b=.04, 95%CI=-.03, .17, p=.25, Latinx b=.07, 95%CI=-.01, .15, p=.08; delta DunedinPACE from age 9 to 15: White b=.27, 95%CI=.18 to .36, p<.001, Black b=.02, 95%CI=-.04, .08, p=.51, LatinX b=-.01, 95%CI=-.09, .07, p=.78). This may be driven by the fact that racially marginalized children are far more likely to live in socioeconomically under resourced neighborhoods (see **Supplemental Material Figure 4**). |
|  | Regress DNAm-aging on neighborhood racial segregation, separately for ages 9 and 15. | See above | See result section and **Supplemental Table 9**. |
|  | Regress DNAm-aging on neighborhood racial segregation, socioeconomic contexts (family level SES and neighborhood disadvantage), and their interaction of neighborhood racial segregation by socioeconomic contexts, separately for ages 9 and 15. | See above | There were no significant interaction effects of SES*neighborhood racial segregation on biological aging measures. |
| To what extent do socioeconomic contexts and police interactions account for racial/ethnic disparities in DNAm-aging? | For significant associations, regress DNAm-aging on children’s race/ethnicity and police interactions. | See above | See **Supplemental Table 8**. We did not see significant association between police interactions and biological aging measures. |
|  | For significant associations, regress DNAm-aging on children’s race/ethnicity and socioeconomic contexts (family level SES and neighborhood disadvantage), |  | See **Supplemental Table 8**. |
| Is skin tone associated with DNAm-aging within racialized youths? | For African-American/Black, Hispanic/Latinx, and Multiracial participants only, regress DNAm-aging at age 9 and 15 on skin tone, race/ethnicity (African-American/Black, Hispanic/Latinx, and Multiracial) and a skin tone x race interaction. |  | See result section, and **Supplemental Table 10**.  There were no significant skin tone*ethnicity/race interactions on biological aging measures. |
| Does  parenting moderate associations of socioeconomic contexts and police  interactions on DNAm  -aging? | For significant associations, regress DNAm-aging on socioeconomic contexts and  parenting. | See above | Associations between parenting and DNAm aging were not significant, see **Supplemental Table 8, 9** and **10**. There were no significant interaction effects for  parenting*SES. |
|  | For significant associations, regress DNAm-aging on police interaction and  parenting. |  | There were no significant interaction effects for parenting*police on biological aging. |
| Does gender moderate associations of race/ethnicity with mental health? | Regress DNAm on children’s race/ethinicty, gender, and an interaction of race by gender. |  | While there was a main effect of sex on DNAm measures (see **Supplemental Table 8**, **9, 10**), there was no significant interaction effect of race by sex on biological aging. |
| Is this association accounted for by covariates? | For significant associations, we rerun analyses adding covariates. | While we did not pre-register to include perinatal covariates, we decided to include them as they are associated with measures of biological aging. | For all significant associations, we ran covariate analyses including postnatal (BMI, smoking, puberty status), and perinatal birth factors (gestational age, birthweight, substance use during pregnancy). See **Supplemental Table 8-10**. |
| Are mental health trajectories across childhood associated with DNAm-aging in adolescence? | Regress DNAm-aging at age 15 on internalizing intercept and slope. |  | See result section and **Supplemental Table 14**. |
|  | Regress DNAm-aging at age 15 on externalizing intercept and slope. |  | See result section and **Supplemental Table 13.** |
|  | Regress DNAm-aging at age 15 on anxiety |  | See result section and **Supplemental Table 15**. |
|  | Regress DNAm-aging at age 15 on depression |  | See result section and **Supplemental Table 15**. |
| Does DNAm-aging at age 9 predict externalizing and internalizing at age 15 and vice versa? | We will fit a fixed-effects regression model to examine the correlation of within-person changes in externalizing and internalizing with changes in DNAm-aging from age 9 to 15 years. | Instead of applying a fixed-effects regression model (FE) and a cross-lagged panel model (CLPM), we applied a latent change model which combines the features of CLPM and FE in one model allowing to more precisely investigate the effect of changes in one variable on changes in the other variable. [1, 2] | See results section **Table 2**, and **Supplemental Table 11** |
|  | We will fit a bivariate random intercept cross-lagged panel model to test whether internalizing and externalizing behavior at age 9 predict DNAm-aging at age 15 and vice versa. | See answer above. | See answer above. |
| Is this association accounted for by covariates? | For significant associations, we rerun analyses adding covariates. | While we did not pre-register to include perinatal covariates, we decided to include them as they are associated with measures of biological aging. | For all significant associations, we ran covariate analyses including postnatal (BMI, smoking, puberty status), and perinatal birth factors (gestational age, birthweight, substance use during pregnancy) as covariates. See results section and **Supplemental Table 11 and 12**. |
| All models  will include age, gender and an age  -by-gender interaction term. |  | We utilized data from a cohort study assessing children at the same age. We therefore only included gender, but not age or age by gender as interaction term in our analyses. |  |

## **Supplemental Material Table 2**

| **Supplemental Material Table 2**  Items included in internalizing and externalizing scales, in line with FFCW codebook (<https://ffcws.princeton.edu/data-and-documentation/scales-and-concepts-documentation>) | |
| --- | --- |
| Age 3 |  |
| Internalizing problems | 16 items, Cronbach Alpha = 0.74 |
| Subscale | Item |
| Anxious depressed | Clings to adults |
|  | Feelings hurt easily |
|  | Too upset by separation |
|  | Look unhappy |
|  | Nervous/high strung |
|  | Self-conscious/easily embarrassed |
|  | Too fearful |
|  | Looks sad |
| Withdrawn | Acts too young for age |
|  | Avoids eye contact |
|  | Doesn't answer when spoken to |
|  | Refuses to participate in games/activities |
|  | Unresponsive to affection |
|  | Shows little affection |
|  | Shows little interest in things |
|  | Withdrawn/doesn't get too involved |
| Externalizing problems | 24 items, Cronbach Alpha= .89 |
| Aggressive | Can't wait turn |
|  | Defiant |
|  | Demanding |
|  | Destroys others' things |
|  | Disobedient |
|  | Does not feel guilty after misbehaving |
|  | Easily frustrated |
|  | Gets in fights |
|  | Hits others |
|  | Hurts animals/people without meaning to |
|  | Angry moods |
|  | Attacks people |
|  | Punishment doesn't change behavior |
|  | Screams a lot |
|  | Selfish/won't share |
|  | Stubborn/sullen/irritable |
|  | Temper tantrums |
|  | Uncooperative |
|  | Wants a lot of attention |
| Destructive | Child can't concentrate, can't pay attention for long |
|  | Child is cruel to animals (also part of agg. Scale) |
|  | Child destroys his/her own things |
|  | Child destroys things belonging to his/her family or other (also part of agg. Scale). |
|  | Child gets into everything |
|  | Child hurts animals or people without meaning to |
|  | Child quickly shifts from one activity to another |
| Age 5 |  |
| Internalizing | 22 items, Cronbach alpha=.75 |
|  | Complains of loneliness |
|  | Cries a lot |
|  | Fears s/he might think/do something wrong |
|  | Feels s/he has to be perfect |
|  | Feels/complains no one loves him/her |
|  | Feels others out to get him/her |
|  | Feels worthless/inferior |
|  | Nervous, high strung or tense |
|  | Too fearful or anxious |
|  | Feels too guilty |
|  | Self-conscious or easily embarrassed |
|  | Suspicious |
|  | Unhappy, sad or depressed |
|  | Worries |
|  | Would rather be alone than with others |
|  | Refuses to talk |
|  | Secretive, keeps things to self |
|  | Shy or timid |
|  | Stares blankly |
|  | Sulks a lot |
|  | Underactive, slow moving, lacks energy |
|  | Withdrawn, doesn’t get involved with others |
| Externalizing | 30 items, Cronbach Alpha = .87 |
| Aggressive behavior | Argues a lot |
|  | Brags or boasts |
|  | Cruel, bullying or mean to others |
|  | Demands a lot of attention |
|  | Destroys his/her own things |
|  | Destroys things belonging to family/others |
|  | Disobedient at home |
|  | Disobedient in school |
|  | Easily jealous |
|  | Gets in many fights |
|  | Physically attacks people |
|  | Screams a lot |
|  | Showing off/clowning |
|  | Stubborn/sullen/irritable |
|  | Has sudden changes in mood or feelings |
|  | Talks too much |
|  | Teases a lot |
|  | Has temper tantrums or hot temper |
|  | Threatens people |
|  | Unusually loud |
| Delingquent behavior | Doesn’t seem to feel guilt after misbehaving |
|  | Hangs around w/ others who get in trouble |
|  | Lies or cheats |
|  | Prefers being with older kids |
|  | Runs away from home |
|  | Sets fire |
|  | Steals at home |
|  | Steals outside home |
|  | Swears or uses obscene language |
|  | Vandalizes |
| Age 9 |  |
| Internalizing | 32 items, Cronbach Alpha .88 |
| Anxious-Depressed |  |
| 13 items | Child cries a lot |
|  | Child fears certain animals, situations, or places, other than school |
|  | Child fears going to school |
|  | Child fears he or she might think or do something bad |
|  | Child feels he or she has to be perfect |
|  | Child feels or complains that no one loves him or her |
|  | Child feels worthless or inferior |
|  | Child is nervous, highstrung, or tense |
|  | Child is too fearful or anxious |
|  | Child feels too guilty |
|  | Child is self-conscious or easily embarrassed |
|  | Child talks about killing self |
|  | Child worries |
| Somatic Complaints | Child has nightmares |
| 11 items | Child is constipated, doesn’t have bowel movements |
|  | Child feels dizzy or lightheaded |
|  | Child is overtired without good reason |
|  | Child has problems with aches/pains without known medical cause |
|  | Child has problems with headaches without known medical cause |
|  | Child has problems with nausea without known medical cause |
|  | Child has problems with eye without known medical cause |
|  | Child has rashes other skin problems without known medical cause |
|  | Child has stomach aches or cramps without known medical cause |
|  | Child has vomiting, throwing up without known medical cause |
| Withdrawn-Depressed | Child enjoys very little |
| 8 items | Child would rather be alone than with others |
|  | Child refuses to talk |
|  | Child is secretive, keeps things to self |
|  | Child is shy or timid |
|  | Child is underactive, slow moving, or lacks energy |
|  | Child is unhappy, sad, or depressed |
|  | Child is withdrawn, doesn’t get involved with others |
| Externalizing | 35 items, Cronbach Alpha .91 |
| Aggressive | Child argues a lot |
|  | Child is cruel, bullies, or shows meanness to others |
|  | Child demands a lot of attention |
|  | Child destroys his or her own things |
|  | Child destroys things belonging to family or others |
|  | Child is disobedient at home |
|  | Child is disobedient at school |
|  | Child gets in many fights |
|  | Child physically attacks people |
|  | Child screams a lot |
|  | Child is stubborn, sullen, or irritable |
|  | Child has sudden changes in mood or feelings |
|  | Child sulks a lot |
|  | Child is suspicious |
|  | Child teases a lot |
|  | Child has temper tantrums or a hot temper |
|  | Child threatens people |
|  | Child is unusually loud |
| Rule-Breaking | Child drinks alcohol without parents’ approval |
|  | Child doesn’t seem to feel guilty after misbehaving |
|  | Child breaks rules at home, school or elsewhere |
|  | Child hangs around with others who get in trouble |
|  | Child lies or cheats |
|  | Child prefers being with older kids |
|  | Child runs away from home |
|  | Child sets fires |
|  | Child has sexual problems |
|  | Child steals at home |
|  | Child steals outside the home |
|  | Child swears or uses obscene language |
|  | Child thinks about sex too much |
|  | Child smokes, chews, or sniffs tobacco |
|  | Child is truant, skips school |
|  | Child uses alcohol or drugs for nonmedical purposes |
|  | Child vandalizes |
| Age 15 |  |
| Internalizing | 8 items, Cronbach Alpha =.79 |
| Anxious/Depressed | Child cries a lot |
|  | Child feels worthless or inferior |
|  | Child is nervous, high-strung, or tense |
|  | Child is too fearful or anxious |
|  | Child feels too guilty |
|  | Child worries |
| Withdrawn | Child is underactive, slow moving, or lacks energy |
|  | Child is unhappy, sad or depressed |
| Externalizing | 20 items, Cronbach Alpha = .89 |
| Aggressive | Child is cruel, bullies, or shows meanness to others |
|  | Child destroys things belonging to the family or others |
|  | Child is disobedient at home |
|  | Child is disobedient at school |
|  | Child gets in many fights |
|  | Child physically attacks people |
|  | Child is stubborn, sullen, or irritable |
|  | Child has temper tantrums or a hot temper |
|  | Child threatens people |
|  | Child is unusually loud |
|  | Child argues a lot |
| Rule-Breaking | Child doesn’t seem to feel guilty after misbehaving |
|  | Child hangs around with others who get in trouble |
|  | Child lies or cheats |
|  | Child runs away from home |
|  | Child sets fires |
|  | Child steals at home |
|  | Child steals outside the home |
|  | Child swears or uses obscene language |
|  | Child vandalizes |

## **Preprocessing DNA data**

DNA extraction and methylation profiling for FFCW was conducted by the Notterman Lab of Princeton University and the Pennsylvania State University College of Medicine Genome Sciences Center. Due to the timing of assay completion 40% of the FFCW saliva samples were completed using the Illumina 450K chip and the remaining 60% used the Illumina EPIC chip. Methods for the two chips were standardized as much as possible, but all analyses were run separately for 450 and EPIC and then meta-analyzed. 450K DNAm image data were processed in R statistical software (4.1) using the Enmix package [3].

The red and green image pairs (n_samples_ =1811) were read into R and the Enmix preprocessENmix and rcp functions were used to normalize dye bias, apply background correction, and adjust for probe-type bias. The majority of sample filtering was applied using the ewastools packages [4]. We dropped samples using the following criteria: if >10% of DNA-methylation sites had detection p-value >0.01 (n_samples_ =34), if there was sex discordance between DNAm predicted sex and recorded sex (n_samples_ =11), or if two sequential samples from the same individual exhibited genetic discordance between visits (n_samples_ =27). ENmix QCinfo function identified samples with outlier methylation values which were cut (n_samples_ =6). Technical replicates were removed n_samples_ =49). This gave us our final analytic sample (n=1684). DNAm sites were removed if they had detection p-value >0.01 in 5% of samples (n=33,376). Relative proportions of immune and epithelial cell types were estimated from DNAm measures using a childhood saliva reference panel [5]. EPIC DNAm image data were processed in R statistical software (4.1) using the ENmix package ( Xu et al., 2016). The red and green image pairs (n_samples_ =2558) were read into R and the ENmix preprocessENmix and rcp functions were used to normalize dye bias, apply background correction, and adjust for probe-type bias. The majority of sample filtering was applied using the Ewastools packages ^7^. We dropped samples using the following criteria: if >10% of DNAm sites had detection p-value >0.05 (n_samples_=63), if there was sex discordance between DNA-methylation predicted sex and recorded sex (n=12), or if two sequential samples from the same individual exhibited genetic discordance between visits (n=30). ENmix QCinfo function identified samples with outlier methylation values which were cut (n=1) or samples that failed bisulfite conversion (n_samples_=7). Technical replicates were removed (n=168). This gave us our final analytic sample (n_samples_=2277). DNAm sites were removed if they had detection p-value >0.05 in 5% of samples (n=127,275). Relative proportions of immune and epithelial cell types were estimated from DNAm measures using a childhood saliva reference panel [5].

## **Supplemental Material Figure 1**


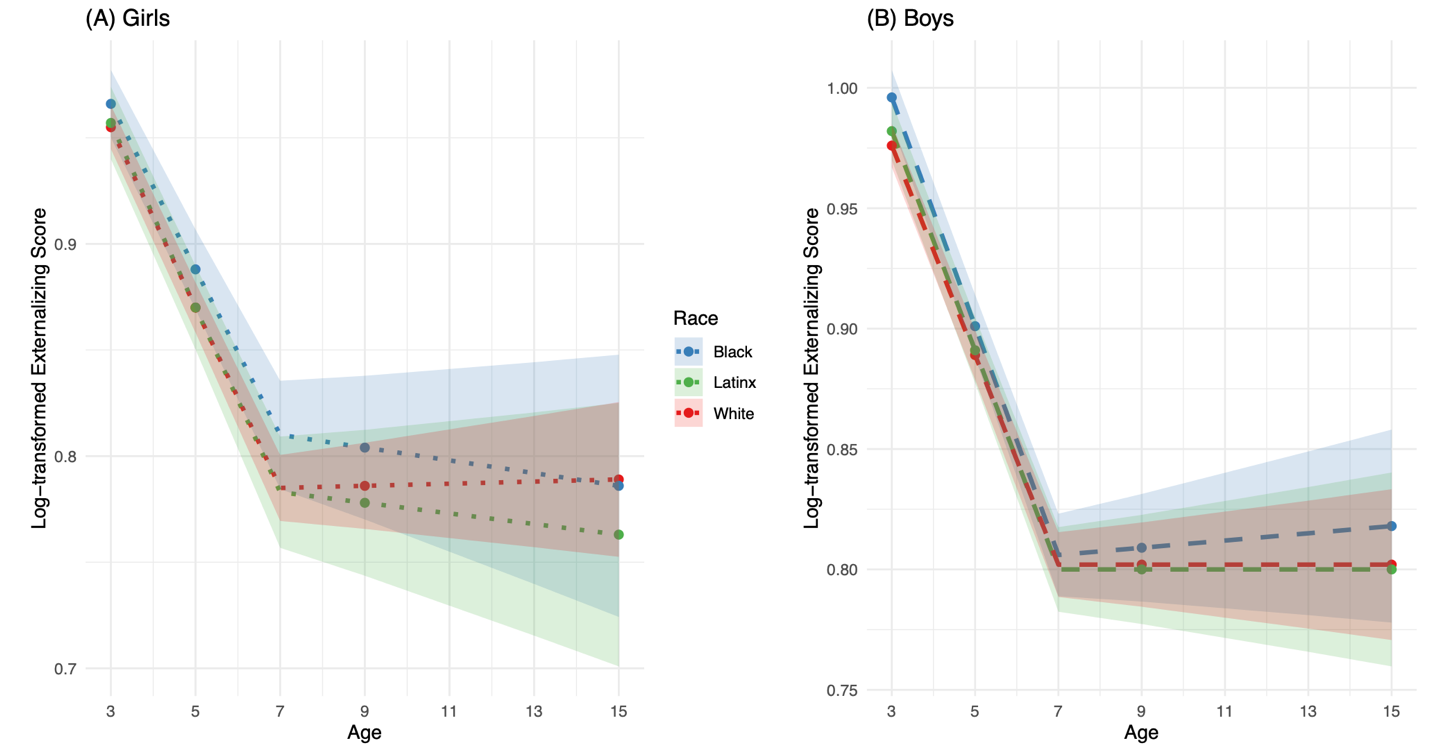


**Supplemental Material Figure 1. Externalizing Scores: Race–Gender Interaction.** Panel **A (Girls)** and Panel **B (Boys)** display the model-predicted mean scores of externalizing behaviors across race and gender groups, including Black, Latinx and White children. Data for the Multiracial and Other groups are not shown for visualization purposes but were included in the analyses. Error bars represent the standard errors of the intercept and slope estimates.

## **Supplemental Material Figure 2**


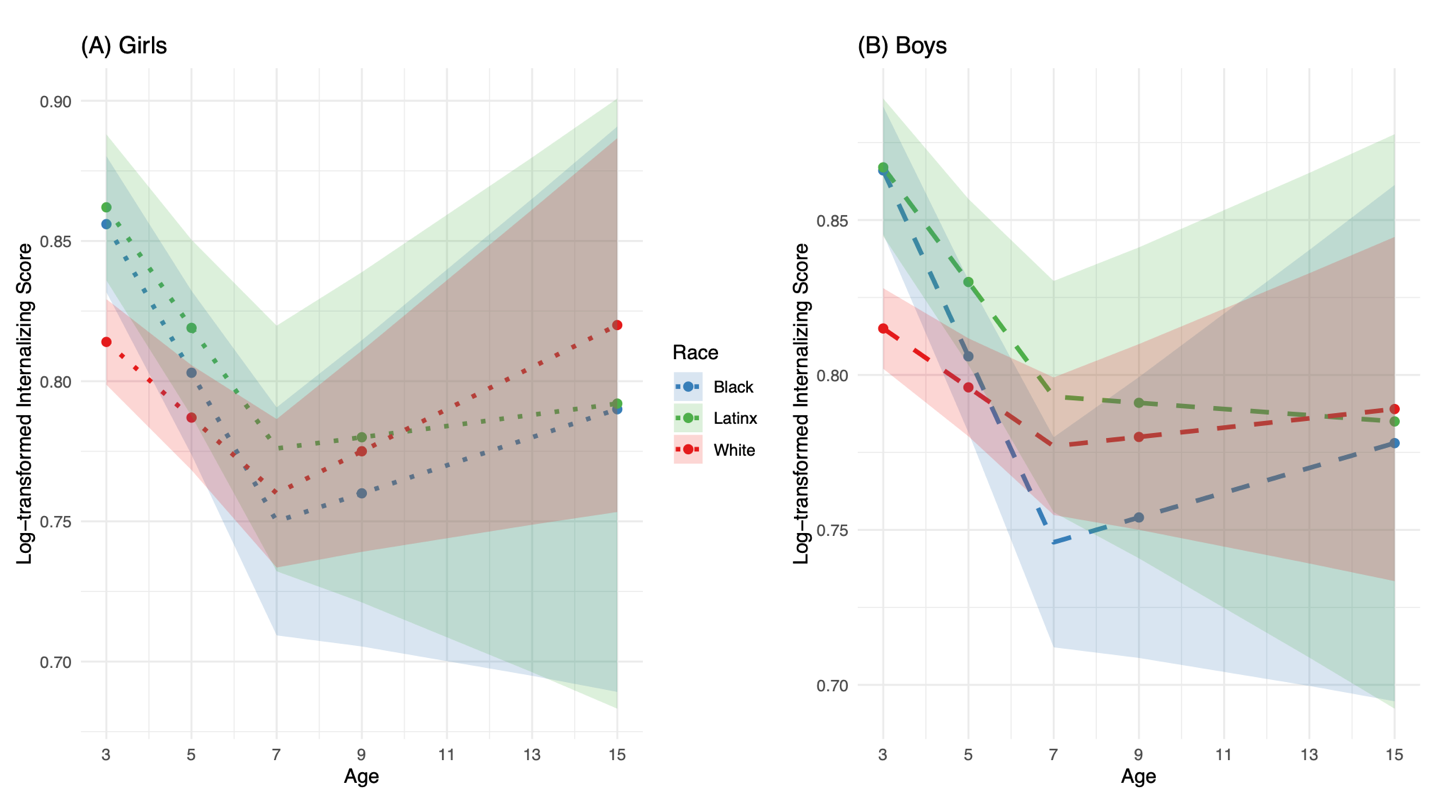


**Supplemental Material Figure 2**. **Internalizing Scores: Race–Gender Interaction**. Panel **A (Girls)** and Panel **B (Boys)** display the model-predicted mean scores of internalizing behaviors across race and gender groups, including Black, Latinx and White children. Data for the Multiracial and Other groups are not shown for visualization purposes but were included in the analyses. Error bars represent the standard errors of the intercept and slope estimates.

## **Supplemental Material Figure 3**

**(A) (B)**


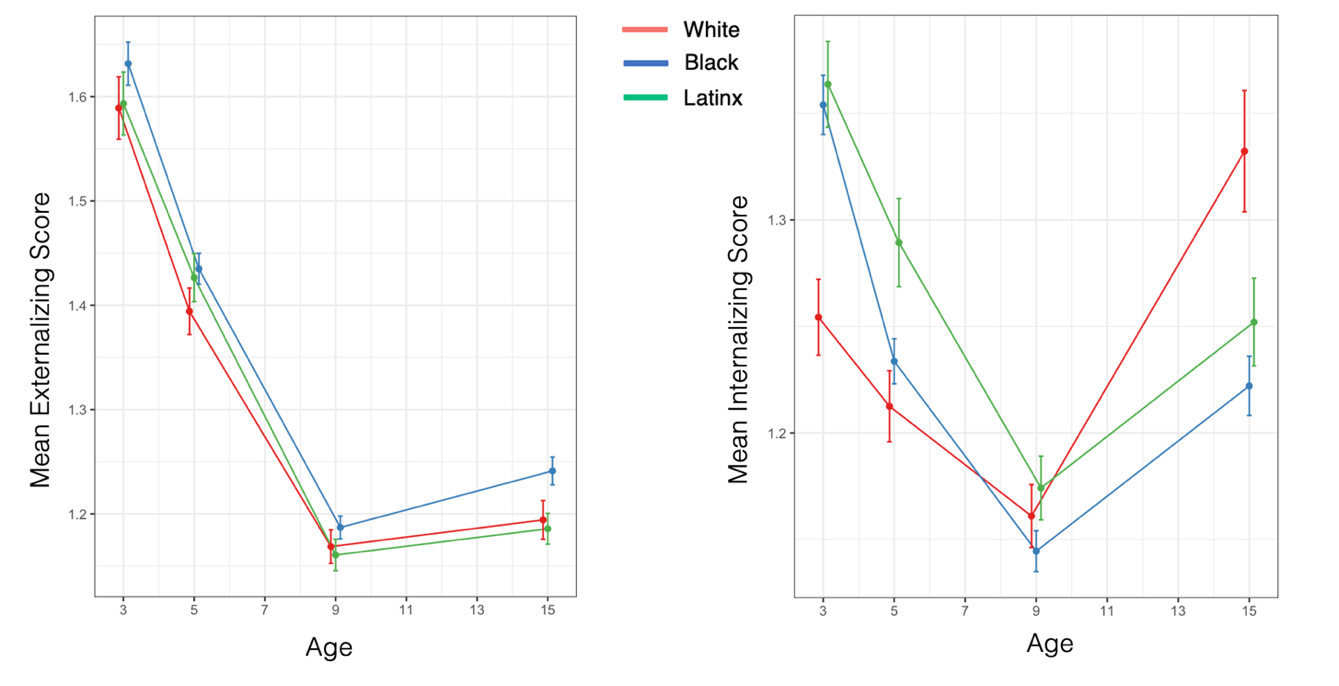


**Supplemental Material Figure 3. Racial and ethnic disparities in internalizing and externalizing behaviors from early childhood through adolescence.** **Panel A** and **B** depict observed mean scores in parent-reported externalizing and internalizing behaviors, respectively, for White, Black, and Latinx children. Data for the smaller subsamples of Multiracial and Other are not shown for visualization purposes but were included in the analyses.

## **Supplemental Material Figure 4**

A)

| 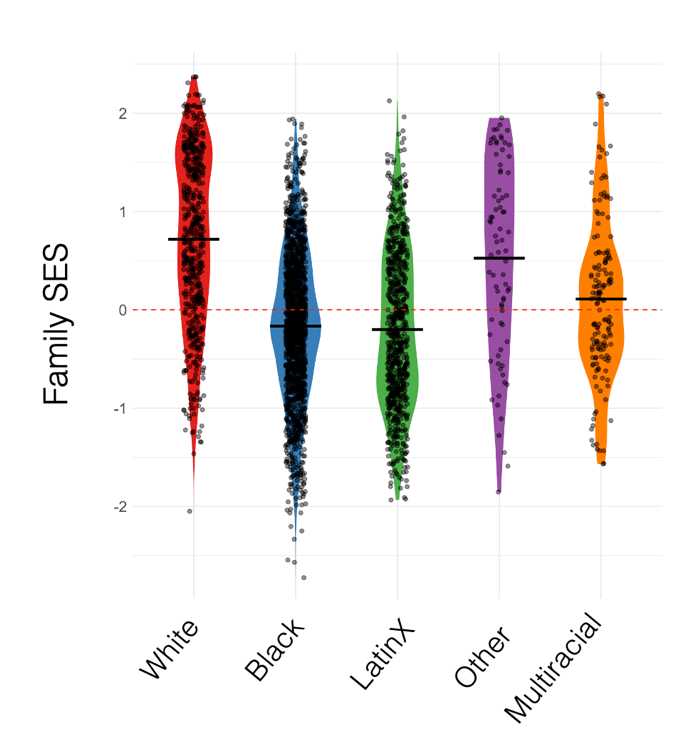  B) C)  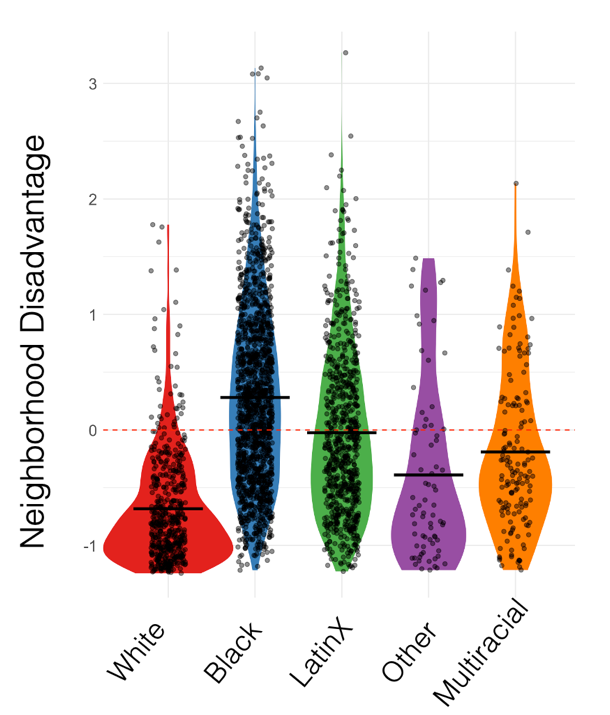 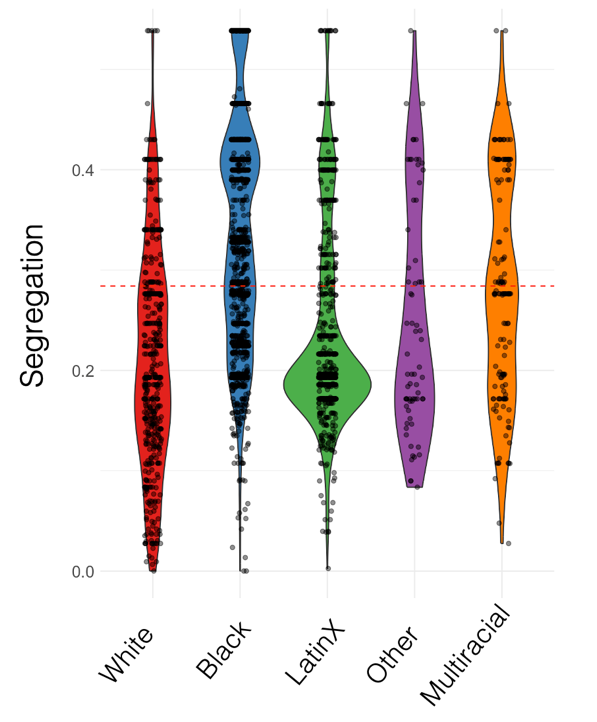 |
| --- |
| **Supplemental Material Figure 4.** **Racial and ethnic disparities in socioeconomic status, neighborhood disadvantage and neighborhood segregation.** **Panel A** plots family socioeconomic status (SES) by racial/ethnic identities, where higher scores indicate higher family SES. **Panel B** plots neighborhood disadvantage by racial/ethnic identities, where higher scores indicate more neighborhood disadvantage. **Panel C** plots neighborhood segregation by racial/ethnic identities, where higher scores indicate more segregation. |

## **Examples of Mplus Scripts**

**Two-slope latent growth curve model (LGCM)**

TITLE: Latent Growth Curve Model exploring Racial Disparities in longitudinal Internalizing Scores from age 3-15

DATA: FILE = "forMplus.dat";

VARIABLE:

NAMES = Int3_log Int5_log Int9_log Int15_log race_black race_hispanic race_other race_multi cm1bsex;

MISSING=.;

VARIABLE:

USEVARIABLES ARE race_black race_hispanic race_other race_multi cm1bsex

MH_3 MH_5 MH_9 MH_15;

DEFINE:

MH_3 = Int3_log;

MH_5 = Int5_log;

MH_9 = Int9_log;

MH_15 = Int15_log;

MODEL:

!initialize latent replacements

MH_7 BY ;

MH_11 BY ;

MH_13 BY ;

! regressions of latents on manifest

I BY MH_3@1.0;

I BY MH_5@1.0;

I BY MH_9@1.0;

I BY MH_15@1.0;

! regressions of latents on latents or manifests

MH_7 ON I@1.0;

MH_11 ON I@1.0;

MH_13 ON I@1.0;

!linear slope 1

S1 BY MH_5@1.0;

S1 BY MH_9@3.0;

S1 BY MH_15@6.0;

!latent replacements

MH_7 ON S1@2.0;

MH_11 ON S1@4.0;

MH_13 ON S1@5.0;

!linear slope 2

S2 BY MH_15@4.0;

S2 BY MH_9@1.0;

!latent replacements

MH_13 ON S2@3.0;

MH_11 ON S2@2.0;

! residuals, variances and covariances

I WITH S1*-0.002 (COV_I_S1);

S1 WITH S2*-0.001 (COV_S1_S);

I WITH S2*0.002 (COV_I_S2);

MH_3*0.006 (E1);

MH_5*0.004 (E2);

MH_7*0.003 (E3);

MH_9*0.003 (E3);

MH_11*0.003 (E3);

MH_13*0.003 (E3);

MH_15*0.003 (E3);

I*0.005 (V_I);

S1*0.001 (V_S1);

S2*0.002 (V_S2);

MH_7 WITH MH_11@0.0;

MH_7 WITH MH_13@0.0;

MH_7 WITH S2@0.0;

MH_11 WITH MH_13@0.0;

! means

[S1*-0.04678](M_S1);

[I*0.8489](M_I);

[S2*0.05918](M_S2);

[MH_3@0];

[MH_5@0];

[MH_7@0];

[MH_9@0];

[MH_11@0];

[MH_13@0];

[MH_15@0];

! mental health associations with race and sex

I ON race_black race_hispanic race_other race_multi cm1bsex;

S1 ON race_black race_hispanic race_other race_multi cm1bsex;

S2 ON race_black race_hispanic race_other race_multi cm1bsex;

ANALYSIS:

TYPE = general;

ESTIMATOR = mlr;

OUTPUT:

sampstat;

cinterval stdyx MODINDICES (ALL);

**Univariate Latent Change Score (LCS) model**

TITLE: Latent Change Score Model exploring race differences in DNAm from age 9 to 15;

DATA: FILE = "forMplus_DNAmOnly_01122023.dat";

VARIABLE:

NAMES = race_black race_hispanic race_other race_multi cm1bsex PCGrim_9_accell_res_array_Std9 PCGrim_15_accell_res_array_Std9 DNAm9 DNAm15

MISSING=.;

Usevariables are race_black race_hispanic race_other race_multi cm1bsex DNAm9 DNAm15 ; !cm1bsex ck6bmip eversmoke Puberty;

DEFINE:

DNAm9 = PCGrim_9_accell_res_array_Std9; !PCPheno_9_accell_res_array_Std9 !PCGrim_9_accell_res_array_Std9; !DunedinPACE_9_res_array_Std9;

DNAm15 = PCGrim_15_accell_res_array_Std9; !PCPheno_15_accell_res_array_Std9 !PCGrim_15_accell_res_array_Std9; !DunedinPACE_15_res_array_Std9;

MODEL:

! regressions of latents on manifest

DIFFdnam BY DNAm15@1.0;

! regressions of manifest on manifest

DIFFdnam ON DNAm9*1.0;

DNAm15 ON DNAm9@1.0;

! regressions of latents on latents or manifests

! residuals, variances and covariances

DNAm9*1.0;

DIFFdnam*1.0;

DNAm15@0;

! means

[DIFFdnam*1.0];

[DNAm9*1.0];

[DNAm15@0];

!associations with race and sex

DIFFdnam ON race_black race_hispanic race_other race_multi cm1bsex;

DNAm9 ON race_black race_hispanic race_other race_multi cm1bsex;

ANALYSIS:

TYPE = general;

ESTIMATOR = ml; !mlr;

OUTPUT:

cinterval stdyx MODINDICES (ALL);

**Bivariate Latent Change Score (LCS) model**

TITLE: Latent Change Score Model association Delta DNAm and Delta MH;

DATA: FILE = "forMplus_DNAmOnly_01122023.dat";

VARIABLE:

NAMES = DunedinPACE_9_res_array_Std9 cm1bsex Ep1 Ep2 Mh1 Mh2; !cm1bsex ck6bmip eversmoke Puberty

;

MISSING=.;

Usevariables are cm1bsex Ep1 Ep2 Mh1 Mh2; !cm1bsex ck6bmip eversmoke Puberty;

DEFINE:

Ep1=DunedinPACE_9_res_array_Std9; !PCPheno_9_accell_res_array_Std9; !DunedinPACE_9_res_array_Std9 !PCGrim_9_accell_res_array_Std9

Ep2=DunedinPACE_15_res_array_Std9; !PCPheno_15_accell_res_array_Std9; !DunedinPACE_15_res_array_Std9 !PCGrim_9_accell_res_array_Std9

Mh1= Int9_log_Std9;

Mh2= Int15_log_Std9;

MODEL:

! for DNAm

! regressions of latents on manifest

DIFFep BY Ep2@1.0;

! regressions of manifest on manifest

DIFFep ON Ep1*1.0;

Ep2 ON Ep1@1.0;

! regressions of latents on latents or manifests

! residuals, variances and covariances

Ep1*1.0;

DIFFep*1.0;

Ep2@0;

! means

[DIFFep*1.0];

[Ep1*1.0];

[Ep2@0];

!associations with covariate sex

DIFFep ON cm1bsex;

Ep1 ON cm1bsex;

! for Mental Health (MH)

! regressions of latents on manifest

DIFFmh BY Mh2@1.0;

! regressions of manifest on manifest

DIFFmh ON Mh1*1.0;

Mh2 ON Mh1@1.0;

! regressions of latents on latents or manifests

! residuals, variances and covariances

Mh1*1.0;

DIFFmh*1.0;

Mh2@0;

! means

[DIFFmh*1.0];

[Mh1*1.0];

[Mh2@0];

!associations with covariate sex

DIFFmh ON cm1bsex;

Mh1 ON cm1bsex;

!covariates between DNAm and MH

DIFFep WITH DIFFmh;

Ep1 WITH Mh1;

DIFFep WITH Mh1;

DIFFmh WITH Ep1;

ANALYSIS:

TYPE = general;

ESTIMATOR = ml;

OUTPUT:

cinterval stdyx MODINDICES (ALL);

!SAVEDATA:

!file is DNAmMH PA EXT.dat;

!SAVE = fscores;

## **References**

1. McArdle JJ. Latent variable modeling of differences and changes with longitudinal data. *Annu Rev Psychol*. 2009;**60**:577–605.

2. Mund M, Nestler S. Beyond the Cross-Lagged Panel Model: Next-generation statistical tools for analyzing interdependencies across the life course. *Advances in Life Course Research*. 2019;**41**:100249.

3. Xu Z, Niu L, Li L, Taylor JA. ENmix: a novel background correction method for Illumina HumanMethylation450 BeadChip. *Nucleic Acids Res*. 2016;**44**:e20.

4. Heiss JA, Just AC. Improved filtering of DNA methylation microarray data by detection p values and its impact on downstream analyses. *Clinical Epigenetics*. 2019;**11**:15.

5. Middleton LYM, Dou J, Fisher J, Heiss JA, Nguyen VK, Just AC, et al. Saliva cell type DNA methylation reference panel for epidemiological studies in children. *Epigenetics*. 2022;**17**:161–177.
